# An Adaptive Information Security System for 5G-Enabled Smart Grid Based on Artificial Neural Network and Case-Based Learning Algorithms

**DOI:** 10.3389/fncom.2022.872978

**Published:** 2022-04-14

**Authors:** Chengzhi Jiang, Hao Xu, Chuanfeng Huang, Qiwei Huang

**Affiliations:** ^1^School of Economics and Management, Nanjing Institute of Technology, Nanjing, China; ^2^School of Information Management, Nanjing University, Nanjing, China

**Keywords:** information security, artificial neural network, case-based learning, smart grid, zero trust

## Abstract

With the deployment of 5G Internet of Things (IoT) in the power system, the efficiency of smart grid is improved by increasing two-way interactions in different layers in smart grid. However, it introduces more attack interfaces that the traditional information security system in smart grid cannot response in time. The neuroscience-inspired models have shown their effectiveness in solving security and optimization problems in smart grid. How to improve the security mechanism in smart grid while taking into account the optimization of data transmission efficiency using neuroscience-inspired algorithms is the problem to be solved in this study. Therefore, an information security system based on artificial neural network (ANN) and improved multiple protection model is proposed. Based on the ANN algorithm, the link state sample space is used to train the model to obtain the optimal transmission path in 5G power communication network. Integrating the intelligent link state module, the zero-trust security protection platform using case-based learning algorithm is designed and taken as the first protection, the network security logical isolation facility is taken as the second protection, and the forward and backward isolation facilities are set as the third protection to achieve the strengthened security of 5G IoT in smart grid. The experimental results show the efficiency and effectiveness of the proposed algorithms. In addition, the experimental results also show that the proposed system can resist malicious terminal access, terminal hijacking, data tampering and eavesdropping, protocol fuzzy, and denial-of-service attacks, so as to reduce the security risks of 5G IoT in smart grid. Since the proposed system can be easily integrated into the existing smart grid structure in China, the proposed system can provide a reference for the design and implementation of 5G IoT in smart grid.

## Introduction

The development of smart grid depends on the intelligent infrastructure to enable a control-feedback loop. With the expansion to distribution side and user load side in the smart grid, the deep integration of 5G technology into the smart grid becomes an inevitable trend (Ma et al., 2021). The 5G technology including 5G network slicing technology can be advantageous in supporting the services of the smart grid such as grid monitoring, precise load control, intelligent distribution automation, and advanced metering infrastructure (AMI) (Matinkhah and Shafik, 2019; Forcan et al., 2020; Liu R. et al., 2021). A 5G communication has the characteristics of high bandwidth, low delay, high reliability, and low power consumption (Zhang et al., 2019). The 5G communication technology has great application potential in scenarios such as enhanced mobile bandwidth, large-scale terminal access, and ultra-low delay communication (Zhang, 2021). Using the advantages of 5G communication technology can not only facilitate the collection and analysis of power consumption data, but also improve the accuracy of power load control. In the power Internet of Things (IoT), building 5G cognitive radio network model and applying it to traditional collection and inspection services can improve the perception and transmission performance of a large number of user nodes (She et al., 2021). The advantages of 5G technology in future smart grid may include that it provides the data acquisition and visualization ability for multiple layers of smart grid (Ahmadzadeh et al., 2021).

At present, the power optical fiber private network communication is mainly used in the power system in China, which has high security and reliability. Due to the limited cost, fiber core resources and mobile operations, it is unable to cover a large number of power business terminals, so that 5G and other wireless communication methods need to be used as a supplement to the optical fiber private network (Wu et al., 2020; Li et al., 2021). However, the 5G networks do not provides end-to-end security for applications in smart grid where new types of threats may be introduced including security misconfiguration at mobile edge computing host (MECH) and IoT device security problems (Borgaonkar and Jaatun, 2019). The critical applications in smart grid requires additional measures against unauthorized access to the network while wireless technology such as 5G is applied (Ghanem et al., 2021). In addition, denial-of-service (DoS) or false data injection attacks may be launched against different parts of AMI using 5G in smart grid, leading to financial losses or even physical damages (Saghezchi et al., 2017). Therefore, the security of power terminal side is very important for the normal operation of power system communication network. Whether the service terminal of power system in China can be safely connected has become an important research direction of researchers in the field of power safety. Meanwhile, to facilitate the deployment of 5G applications, the security measures need to be easily integrated into the existing power industry security protection strategies (Li et al., 2020).

The current research on 5G IoT in smart grid mainly focuses on meeting different business needs, improving business processing efficiency and network scalability. In terms of security protection, it is mainly based on the existing security protection strategies and equipment that can no longer meet the security requirements in the IoT and 5G era. Therefore, to strengthen its security protection mechanism while improving the efficiency of 5G IoT, this study proposes an improved information system based on ANN and improved multiple protection mechanism, which can be easily integrated into the existing smart grid security architecture. The proposed method evaluates, learns, and predicts the link states in the process of 5G power communication (Hu et al., 2019), and adopts the multiple security protection method in combination with the idea of double isolation power security access area (Cao et al., 2019a) and the encryption, authentication method (Zhao, 2020) to improve the transmission efficiency of power 5G communication while meeting the security requirements in the process of power 5G communications.

## Related Work

Scholars in related fields have studied the power communication access scheme and achieved some research results. Li et al. (2018) designed an intelligent power distribution terminal access architecture based on the integration of multiple technologies such as wireless sensor network (WSN), wireless local area network (WLAN) and wired private network, and adopted data hierarchical encryption, access network security classification and isolation to ensure network security. The architecture can effectively meet a variety of business needs of power distribution terminals. Chen et al. designed a joint deployment architecture based on multi-access edge computing (MEC), and designed a task scheduling mechanism by deploying MEC network elements on the access side and the core network side (Chen et al., 2018). The deployment architecture can effectively allocate MEC processing nodes and effectively improve scalability. Saghezchi et al. proposed a security architecture incorporating intrusion detection system (IDS) into AMI to protect the integrity of the information exchanged (Saghezchi et al., 2017).

The neuroscience-inspired methods [including artificial neural network (ANN)] have shown the effectiveness in solving security and optimization problems in smart grid. To mitigate the false data injection attacks in smart grid, the graph neural network (GNN) based detector incorporating physical connections and exploiting spatial correlations (Boyaci et al., 2022) or the detector combining predictions of Kalman filter and recurrent neural network (RNN) (Wang et al., 2022) can be effective methods. The RNN can also be applied to classify multiclass attacks for power systems with high accuracy (Hong et al., 2020). In addition, neuroscience-inspired methods can be applied to optimization problems in smart grid such as link quality estimation in smart grid WSN (Sun et al., 2017), load monitoring (Zhou et al., 2022), short-term load forecasting (Deng et al., 2021), and power user behavior feature classification (Deng et al., 2022).

As the organizational boundaries have become blurred, the zero-trust architecture has been attracting information security researches and is expected to be further explored and implemented in future digital systems (Wylde, 2021). The power grid security architecture can be established based on zero-trust architecture to provide dynamic security policies according to the trust of the access entities (Liu T. et al., 2021). The specific implementation of zero-trust architecture is considered as the improvement on continuous risk management. The intelligent decision support system using case-based reasoning (CBR) and rule-based machine learning may be used to significantly reduce the risks in software development (Asif and Ahmed, 2020).

Inspired by the adaptive ability and effectiveness of neuroscience-inspired methods and zero-trust models in the above researches, we attempt to design algorithms using ANN and case-based learning to improve the security and communication efficiency in 5G IoT environment of smart grid.

## System Model

**Figure 1:**
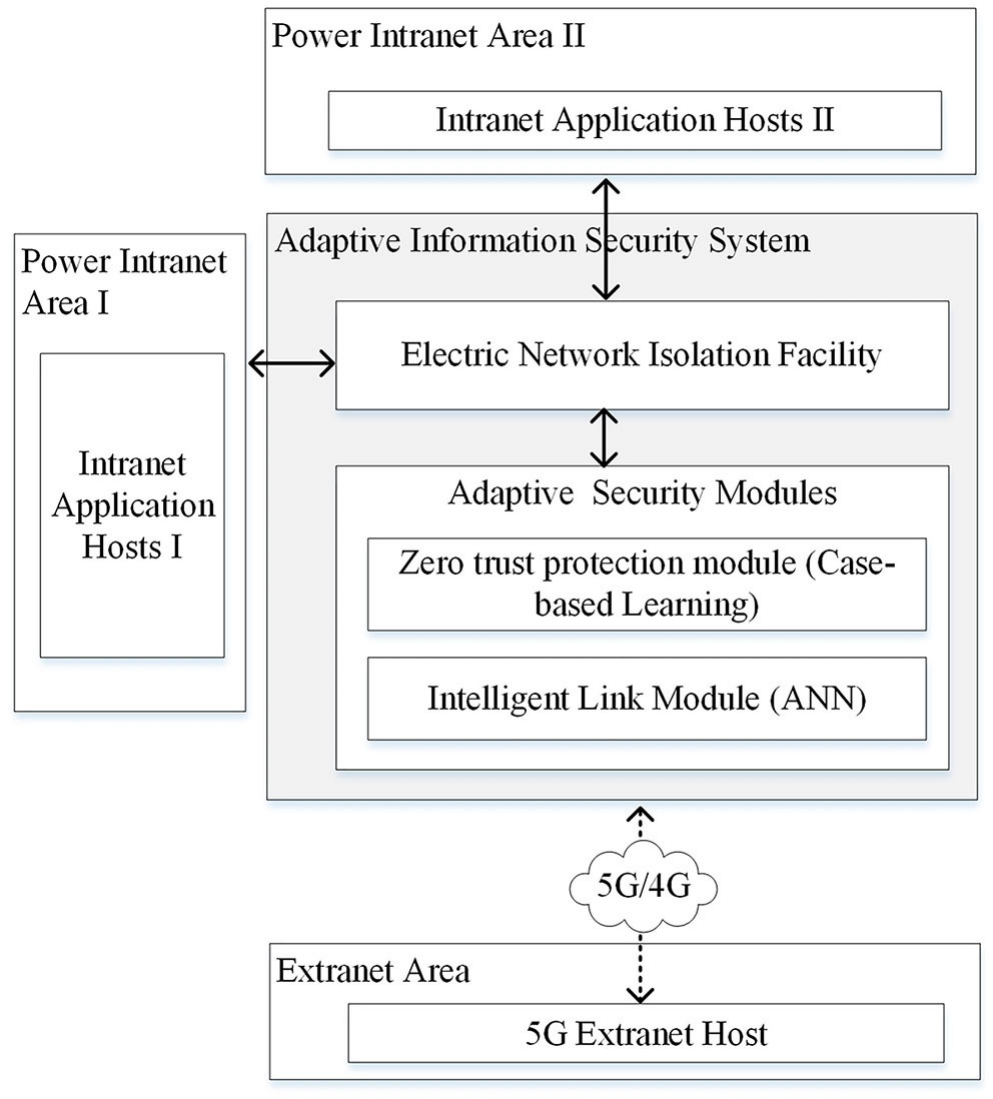
Adaptive information security system model.

According to the power security regulations and current implementation of smart grid information infrastructure in China, an information security system model is proposed as shown in Figure 1.

As shown in Figure 1, the adaptive information security system is implemented in the secure access area in the power information network, which consists of a zero-trust protection module based on case-based learning and an intelligent link module based on ANN. The details of those two main components will be described in Sections Related Work and System Model. The power intranet area I represents the network area where power production and control related software and hardware are implemented such as supervisory control and data acquisition (SCADA) and energy management system (EMS). Servers and equipment running these applications are represented by intranet application hosts I. The power intranet area II represents the network area where power management related data is processed such as office automation (OA) and enterprise resource planning (ERP). Servers and equipment running these applications are represented by intranet application hosts II. The power intelligent terminal and other equipment that implement in-field monitoring or control functions *via* public network such as 5G/4G, narrow band internet of things (NB-IoT), and long range radio (LoRA) can be represented by 5G extranet host.

**Figure 2:**
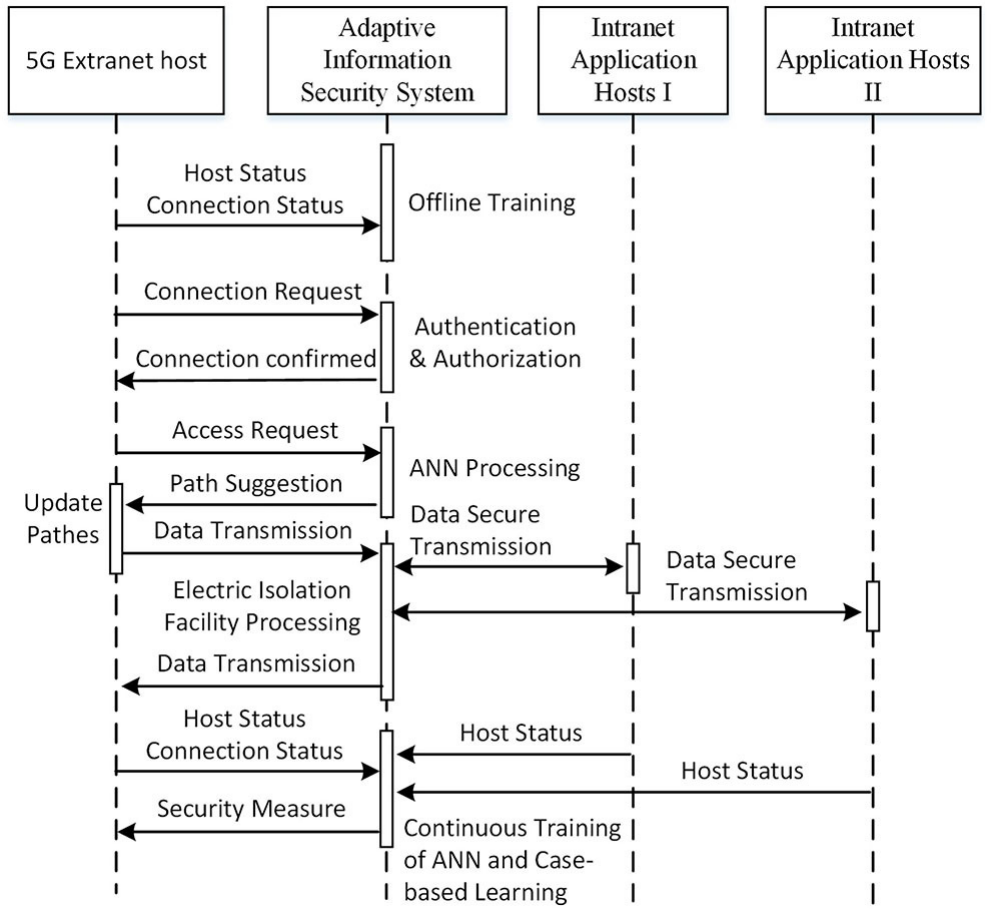
Overall process of the system.

Since the security level and requirements of power intranet area I and II are different, customized security policies and measures should be made. The overall secure communication process is shown in Figure 2.

It can be seen from Figure 2 that the initialization of the proposed system is completed by offline training while acquiring status of connections and hosts for a period. First, 5G extranet host initiates a connection request to proposed system that verifies the identity of 5G extranet host. If the identification process succeeds, the appropriate authority is configured to extranet host. Then, 5G extranet host sends a request for data transmission path with its status and requested time slot. The proposed system produces a suggested path for extranet host and the data transmission is processed. The states of links between the proposed system and 5G extranet host are updated periodically so that an up-to-date suggested path can be produced by the proposed system. The security risks in intranet and extranet are continuously monitored by the proposed system and the credibility of each active user in the network is evaluated accordingly. The authority for each active user may be adjusted according to its real-time credibility so that the multiple security protection is strengthened.

The following sections will describe the intelligent link state module and improved multiple protection model in details.

## Intelligent Link Module Based on ANN

In the intelligent link module, an ANN algorithm is applied to design an adaptive routing algorithm to obtain the link states in 5G communication network. The 5G and 4G communication modes are both supported in the communication network. Through the forward conduction and the backward conduction, the deep neural network operation is completed (Liu et al., 2020).

Suppose there is a 5G power communication network with *N* nodes, {*D*_1_, *D*_2_, ⋯ , *D*_*N*_} represents the node set. The loads of the network nodes are collected during the collection time period Δ*t* of the cognitive plane, while the packet loss rates of the transmission paths from the source node to the target node are calculated. According to the transmission performance requirements of power services, the packet loss rates of the transmission paths are divided into four categories from low to high as {0: Ultra low; 1: Low; 2: Average; 3: High}. We can use $l=\left({\overset{⇀}{x}}_{i,j},ts_{i,j},y_{i,j}\right)$ as a data sample where ${\overset{⇀}{x}}_{i,j}\;=\;\left\{\;D_{i},D_{i+1},\ldots ,D_{j}\right\}$,*ts*_*i,j*_ represents the collection time span, *y*_*i,j*_ represents the categorized packet loss rate from node *i* to node *j*, and *y*_*i,j*_ ∈ {0, 1, 2, 3}. Hence, the sample space including the data label *y*_*i,j*_ is represented as follows:


(1) $$\begin{array}{l} Y=\{\left({\vec{x}}_{i,j,1},ts_{i,j,1},y_{i,j,1}\right),\left({\vec{x}}_{i,j,2},ts_{i,j,2},y_{i,j,2}\right),\\ \;\cdots ,\left({\vec{x}}_{i,j,n},ts_{i,j,n},y_{i,j,n}\right),\cdots \} \end{array}$$

The forward conduction that outputs link state prediction value is completed based on the non-linear function formed by each layer node in the deep neural network. The forward conduction expression is as follows:


(2) $$\begin{array}{l} Y_{\left(l,k\right)}\left(x\right)=F\left(\overset{n}{\underset{i=1}{\sum }}\left(w_{i,k}^{l}\times x_{i}+b_{i}^{l}\right)\right) \end{array}$$

where *k* = 1, 2, ⋯ , *n*, $w_{i,k}^{l}$ represents the weight from neuron *k* of layer (*l*+1) to neuron *i* of layer *l*, *F* and *w* denote the non-linear function and the weight matrix, respectively, and $b_{i}^{l}$ represents the bias of neuron *i* of layer *l*.

The loss function is used to express the error between the sample space and the output value of neural network. The loss function is shown as follows:


(3) $$\begin{array}{l} J\left(w,b;x,y\right)=\frac{1}{2n}{\overset{n}{\underset{i=1}{\sum }}\|Y\left(w,b,x^{i}\right)-y^{i}\|}^{2} \end{array}$$

where *b* represents the square loss, *x*^*i*^ represents the absolute value loss, and *w* represents the mean square error loss.

The gradient descent method is selected to reduce the error between the calculated sample value and the predicted value. Using the gradient descent method and step-by-step iterative solution, the predicted value of link state, the minimum value of sample space loss function and model parameters can be obtained after completing the backward conduction. The backward function is shown as follows where *i* = *l* − 1.


(4) $$\begin{array}{l} \delta^{i,l}={\left(w^{l+1}\right)}^{T}\times \delta^{i,l+1}\times {Y^{\prime }}_{\left(i,l\right)} \end{array}$$

The updating formulas of *w* and *b* are shown as follows:


(5) $$\begin{array}{l} w^{l}=w^{l}-\alpha \overset{n}{\underset{i=1}{\sum }}\delta^{i,l}\times {\left(Y_{i,l-1}\right)}^{T} \end{array}$$

(6) $$\begin{array}{l} b^{l}=b^{l}-\alpha \overset{n}{\underset{i=1}{\sum }}\delta^{i,l} \end{array}$$

where α represents the iteration step. We can set the threshold value as ε. When the updated value of *w* and *b* are less than the threshold value, the calculation will be terminated. Input the test set samples into the model, and count the error between the model output results and the sample values. Repeating the above process until the error is lower than the predefined threshold, the accuracy test is completed.

The ANN algorithm can be applied to 5G power communication with complex changes, and output the results most consistent with the current environment according to the real-time change of link state in the network (Ge et al., 2020). The link state sample space is input into the model. After the model passes the hidden layer operation, select the softmax function to apply to the output layer, output the probability value of each path (Zhu et al., 2020).

The application plane includes network applications such as routing and network virtualization. The cognitive plane is composed of switches and other devices, and the control plane refers to the controller in the logic set. After receiving the service request sent by the control plane, the application plane forwards it to the cognitive plane. When the output path of the cognitive plane is still the original path, the decision information is set according to the initial routing information table. When the output path of the cognitive plane changes, the new transmission path is sent back to the control plane, and the routing information table is updated by the control plane in real time.

After a fixed interval, the control plane needs to reset the network route. It updates the routing table information in real time (Xu et al., 2018) and transmits the updated routing table to the control layer that controls the cognitive plane to retrain the model, and updates the model in real time after training. Through the above steps, an adaptive routing algorithm is designed using neural network model. Through the forward conduction and the backward conduction, the deep neural network operation is completed to obtain the optimal transmission path in 5G communication network.

## Improved Multiple Protection Model Base on Case-Based Learning

**Figure 3:**
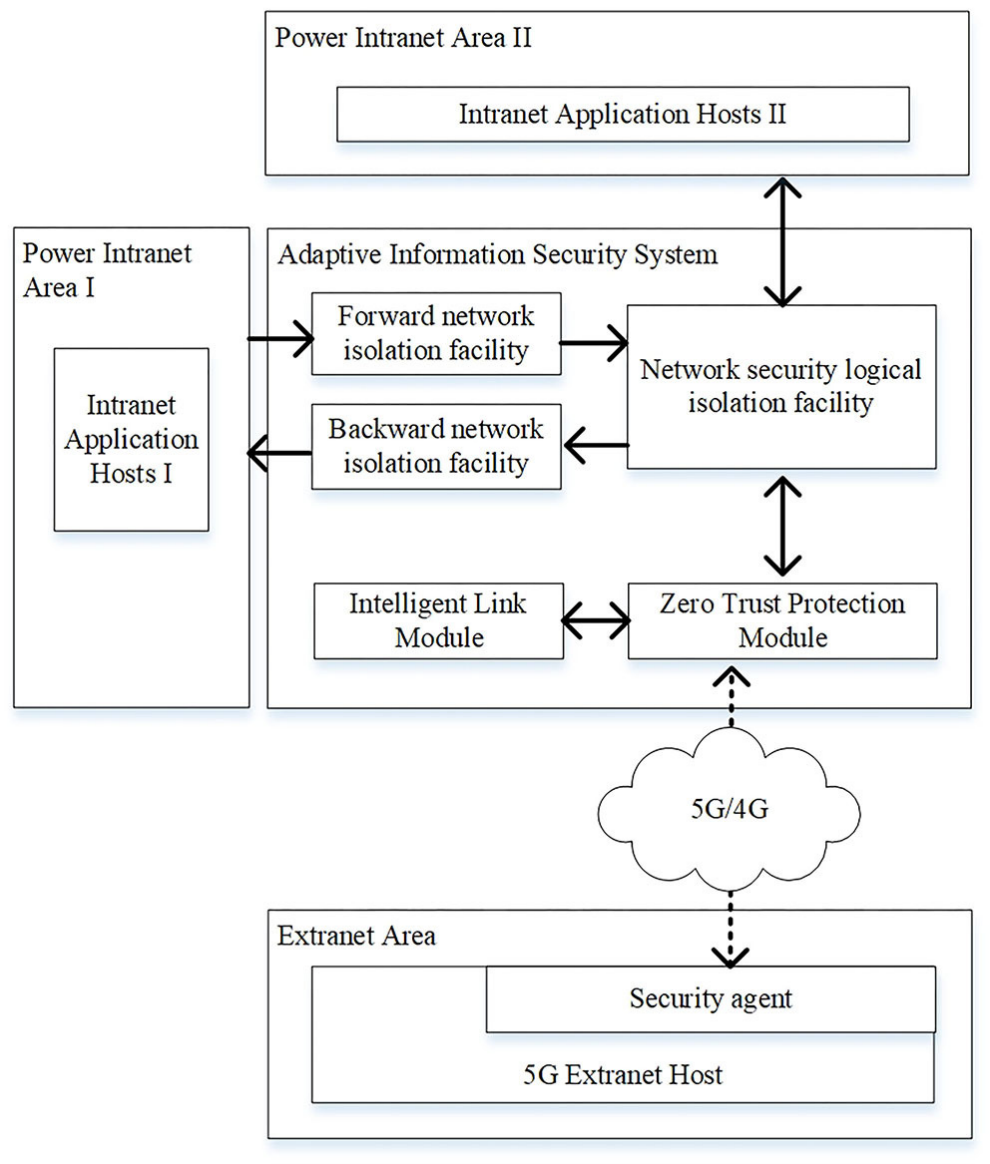
Improved multiple protection model.

As shown in Figure 3, the improved multiple protection model is composed of the zero-trust protection module, network security logical isolation facility, forward and backward network isolation facility. At present, the power terminals mainly focus on the realization of business functions, and their security functions generally are not fully considered. They need to be improved in terms of access authorization, audit, and network attack protection (Cheng et al., 2020). Therefore, the zero-trust protection module in the secure access area not only serves as a boundary isolation facility, but also carries out continuous trust and risk assessment for 5G external network hosts. The zero-trust protection module integrates the lightweight encryption and authentication center that uses the identity-based cryptosystem (IBC) or combined public key (CPK) system to generate and distribute the keys to the 5G external network hosts. The network security logical isolation facility in the security access area mainly implements the gate isolation function and the power protocol data security filtering function (Han et al., 2019). The forward and backward network isolation facilities in the secure access area use the existing devices or use the enhanced forward and backward isolation devices (Cao et al., 2019b).

The zero-trust architecture can provide active defense ability and end-to-end security enforcement in a 5G smart application environment where a four-dimensional framework may be designed including subject, object, environment, and behavior (Chen et al., 2021). In power industry of China, the credit management and risk assessment are also paid attentions, considering the risks in the power market transactions (Cai et al., 2020). Thus, a CBR algorithm is proposed in the zero-trust protection module to implement the continuous credit and risk management.

The operational process of improved multiple protection model includes the following steps:

Step 1. The 5G extranet host establishes a network connection with the zero-trust protection module that verifies its identity information. If it is a legal terminal, an encrypted transmission channel is established and access rights are configured. The 5G extranet host requests the optimal path from the 5G communication link optimization service that then returns the result after calculating the predicted value of the optimal path. The zero-trust protection module evaluates the trust and risk value of the extranet host by monitoring the status and the behavior of the extranet host in real time, and adjusts the access authority of the extranet host according to the CBR algorithm that will be described later in this section.
Step 2. After receiving the cipher text sent by 5G power communication network, the zero-trust protection module decrypts the cipher text and transmits the plaintext to the network security isolation facility.
Step 3. After receiving plaintext data, the network security isolation facility implements network protocol stripping (Wang, 2018), and performs security filtering on the obtained data based on pattern matching and feature filtering methods. The plaintext is then signed after security filtering. If the extranet host needs to access the service application of Intranet area II, go to Step 4. If the extranet host needs to access the service application of Intranet area I, go to Step 5.
Step 4. Send the signed data to the access gateway of Intranet area II, encapsulate the network protocol and send the message to the intranet application host II, and the communication of Intranet area II host ends.
Step 5. Convert the signed data into private protocol message of backward isolation facility and output it to backward isolation facility.
Step 6. After receiving the private protocol message, the backward isolation facility performs signature verification, data content filtering and validity check, and sends the data to the access gateway of Intranet area I.
Step 7. The access gateway of Intranet area I encapsulates the data with network protocol and sends it to the intranet application host I, and the communication of Intranet area I host ends.
Step 8. The communication process between the intranet host and the extranet host is opposite to the above process.

**Figure 4:**
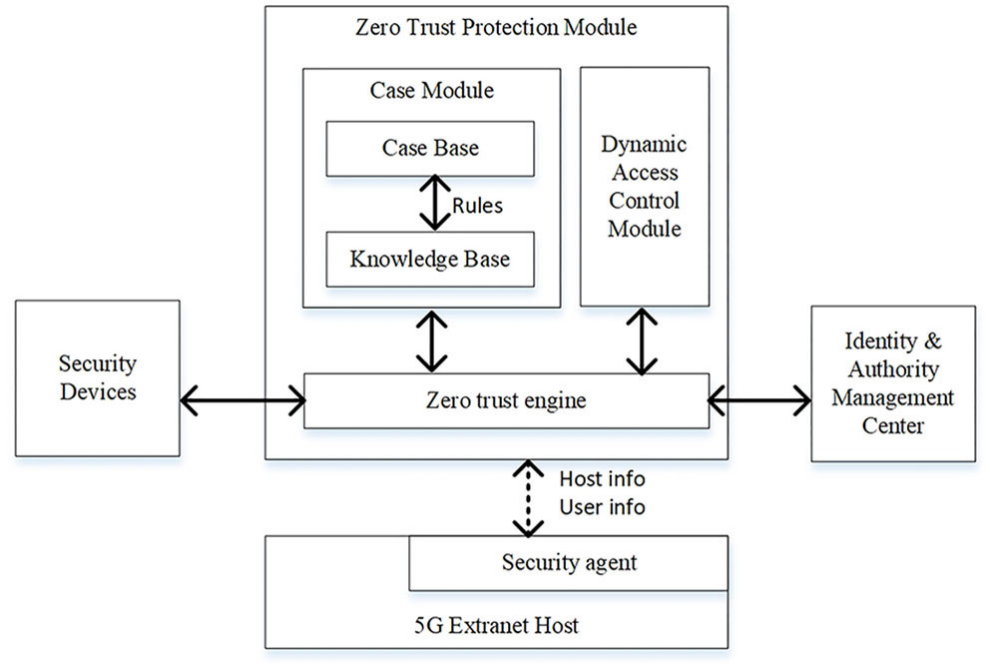
Architecture of zero trust protection module.

The architecture of zero-trust protection module is shown in Figure 4.

As shown in Figure 4, the zero-trust security platform is comprised of a case module, a dynamic access control module and a zero-trust engine. The operational process of the platform will be described following the design and implementation of CBR method. The CBR method is a recycling process including five phases: modeling, search, reuse, review, and retain (Chourib et al., 2020). Each case (*case*_*i*_) in the case base of case module is modeled in Formula (7).


(7) $$\begin{array}{lll} case_{i}=\left\{caseID_{i},state_{i},event_{i},credit_{i},group_{i}\right\}\\ state_{i}=\left\{ID\text{\_}E_{i},ID\text{\_}U_{i},type_{i},biz_{i},ip_{i}\right\}\\ event_{i} & \in & \left\{time_{i},freq_{i},tgt_{i},vol_{i},cfg_{i},warn_{i},usre_{i}\right\} \end{array}$$

$$\begin{array}{lll} credit_{i} & \in & \left\{cred_{i,1},cred_{i,2},\ldots ,cred_{i,n}\right\}\\ effe_{i} & \in & \left\{e_{i,1,2},e_{i,2,3},\ldots e_{i,n-1,n}\right\}\\ group_{i} & \in & \left\{CaseID_{1},\ldots ,CaseID_{i-1},CaseID_{i+1},\ldots CaseID_{n}\right\} \end{array}$$

where *state*_*i*_ represents the status of 5G extranet host in *case*_*i*_ where *ID*_*E*_*i*_ is the unique identity name of the 5G extranet host if IBC system is adopted or the equipment certificate otherwise. Here, *ID*_*U*_*i*_ is the user identity certificate in the host, *type*_*i*_ is the type of the host, *biz*_*i*_ is the power business running in the host, and *ip*_*i*_ is the IP address of the corresponding host. Also, *event*_*i*_ represents the events encountered that may be the abnormal behaviors in terms of data transmission time (*time*_*i*_), data transmission frequency (*freq*_*i*_), data transmission target (*tgt*_*i*_), data transmission volume (*vol*_*i*_), configuration change (*cfg*_*i*_), and user defined event (*usre*_*i*_). Now, *credit*_*i*_ is the credits record of the last *n* credits of *ID*_*E*_*i*_ and *ID*_*U*_*i*_. records the effectiveness evaluated for each change in *credit*_*i*_. Lastly, *group*_*i*_ represents the set of case IDs related to *case*_*i*_.

The knowledge base is comprised of power business templates and rule sets as defined in Formula (8).


(8) $$\begin{array}{l} Template_{i}=\left\{bizID_{i},bizText_{i},time_{i},freq_{i},tgt_{i},vol_{i}\right\}\\ Rule_{default}=\left\{bizID,event,mea_{default}\right\} \end{array}$$

$$\begin{array}{l} Rule_{i}=\left\{event_{i},mea_{i}\right\} \end{array}$$

where *Template*_*i*_ represents a template for a specific power business. Here, *bizID*_*i*_ and *bizText*_*i*_ denote the identity number and description of a power business, respectively. Also, *time*_*i*_, *freq*_*i*_, *tgt*_*i*_, *vol*_*i*_ are the data transmission time, frequency, target, and volume, respectively, defined by business personnel based on the regular operations of business applications. Now, *Rule*_*default*_ defines the default measures when an event occurs in a business application environment. Also, *Rule*_*i*_ represents a rule defined in the rule sets that decides which measures in *mea*_*i*_ can be adopted when an event in *event*_*i*_ occurs. Therefore, the recycling five phases in CBR may include the following steps:

Step 1. Modeling. The case to be solved can be modeled as {*sta*_*i*_, *evt*_*i*_}. *evt*_*i*_ can be collected by zero-trust engine from security facilities such as IDS, firewall, and UTM. Then, the *sta*_*i*_ can be collected by zero-trust engine from security agents installed in related extranet host and the identity and authority management center.
Step 2. Search. First, *sta*_*i*_ and *evt*_*i*_ are searched in the case base. If *sta*_*i*_ ∈ *case*_*i*_ AND *evt*_*i*_ ∈ *case*_*i*_, go to Step 3.1. If *sta*_*i*_ ∉ *case*_*i*_ AND *evt*_*i*_ ∈ *case*_*i*_, go to Step 3.2. Otherwise, go to Step 3.3.
Step 3. Reuse. The information and knowledge from similar case are used to form the solution for encountered case.
  Step 3.1. If the *e*_*i,n* − 1, *n*_ in *effe*_*i*_ is positive, reuse the last credit change measure (*cred*_*i,n*_ − *cred*_*i,n*−1_} in *credit*_*i*_ of *case*_*i*_. Go to Step 4. Otherwise, go to Step 3.3.
  Step 3.2. If *type*_*i*_, *biz* in *sta*_*i*_ equals to *type*_*i*_, *biz* in *state*_*i*_ of *case*_*i*_, go to Step 3.1. Otherwise, go to Step 3.3.
  Step 3.3. Execute *Rule*_*default*_.
Step 4. Review. The zero-trust engine collects the information from security devices to evaluate the effectiveness of the reused solution.
Step 5. Retain. Update *case*_*i*_ or add a new case to the case base.

In summary, the improved multiple protection model implements the triple security protection from the following aspects:

1. The zero-trust protection module in the secure access area implements the first boundary security isolation. The zero-trust security protection platform monitors the data access, configuration update, and other behaviors of 5G extranet hosts in real time, dynamically evaluates the security risks of 5G extranet hosts and controls the dynamic access rights. The advantage of using zero-trust protection module that integrates lightweight encryption and authentication module is that it reduces the computing capability requirements of 5G external network host, and can continuously monitor and control the security of 5G terminals, which can effectively reduce the access risk of extranet host. The zero-trust protection module avoids the security risk caused by the traditional one-time authorization and permanent effectiveness so as to improve the traditional security model.
2. As the second protection of the model, the network security logical isolation facility implements data security filtering and network logic isolation, and ensures the encryption and authentication of data interaction between the zero-trust security protection platform and the network security isolation facility.
3. As the third protection of the model, the forward and backward isolation facilities are used to block the TCP connection, control the information flow access process, and implement the content filtering in the communication process.

## Experiments and Results Analysis

**Figure 5:**
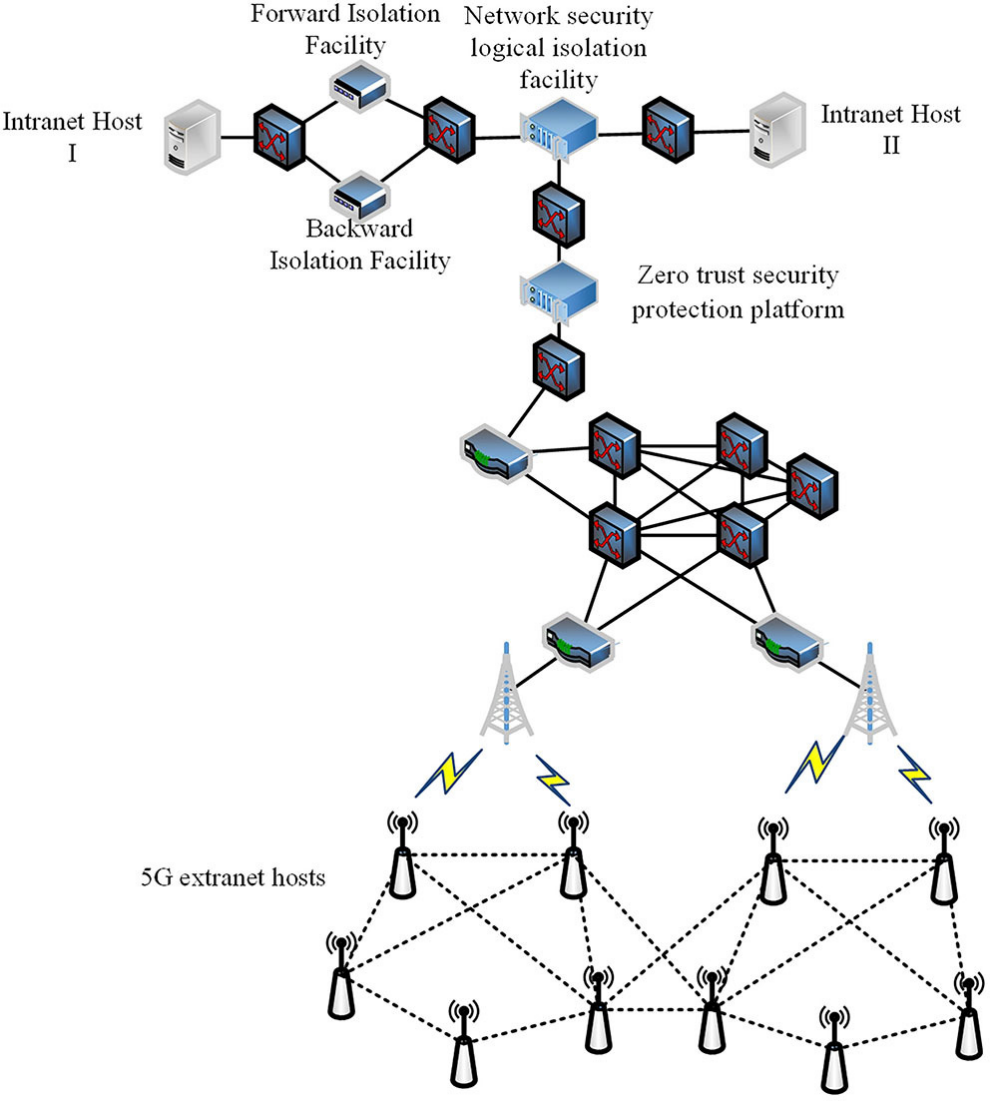
Test environment.

To verify the improvement of communication efficiency and the network security of the proposed system based on ANN and multiple protection model, a test and verification environment combining virtual and reality based on OPNET and security equipment is built as shown in Figure 5. The environment is implemented in an experimental 5G power IoT scenario of State Grid Corporation of China (SGCC).

**Figure 6:**
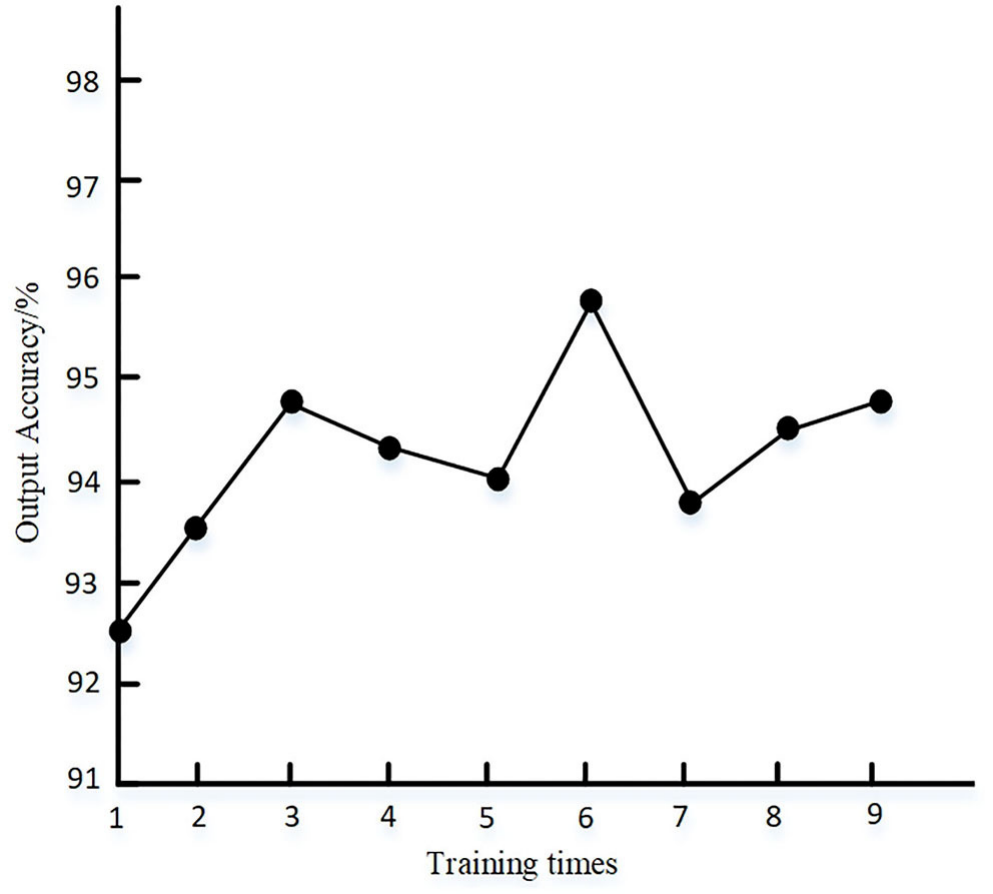
Accuracy test of deep learning method.

First, through experiment 1, the effectiveness and efficiency of the link state algorithm are verified. The dataset is acquired in experimental 5G power IoT scenario for 2 days from Friday to Saturday and then it is marked manually. The ratio of training set to test set of deep learning network is 7:3, and the number of nodes in input layer and output layer are set to 19 and 4, respectively. The full connection mode is adopted, and the softmax function is set as the activation function of the model. The number of iterations and learning rate are 1,000 and 0.1, respectively. The statistical results of deep learning output accuracy under different training times are shown in Figure 6.

**Figure 7:**
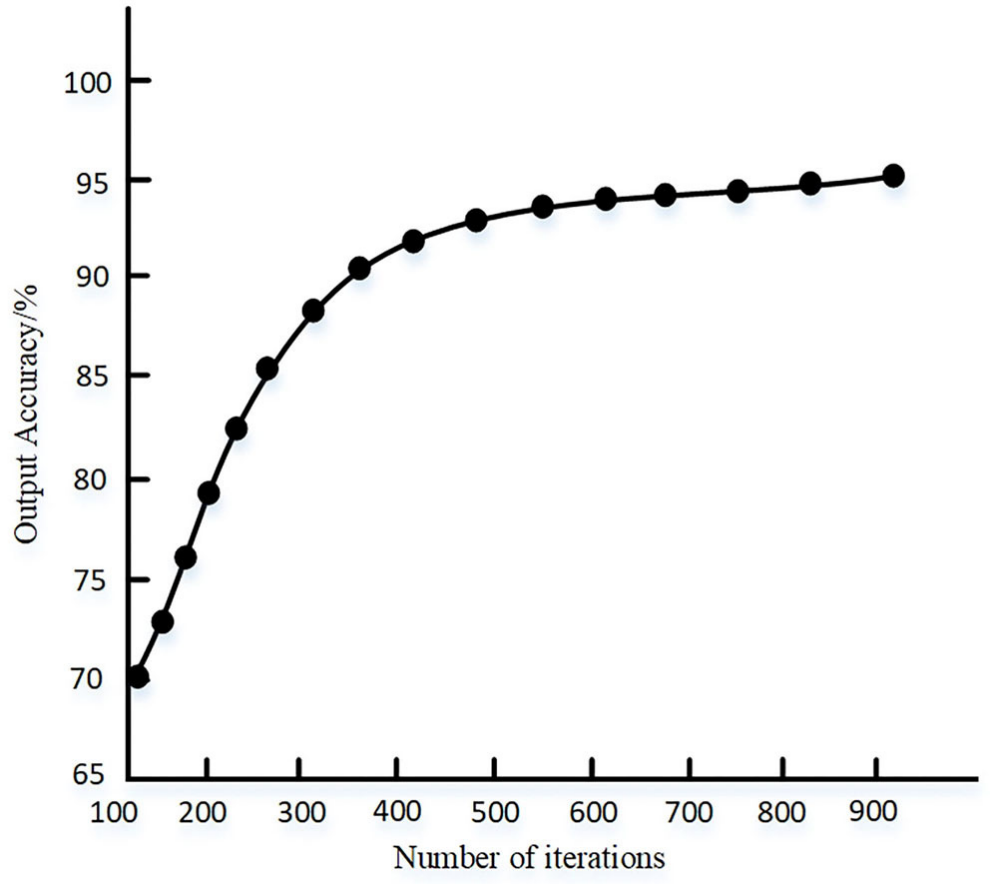
Convergence performance of the algorithm.

It can be seen from Figure 6 that the output accuracy of the proposed method is higher than 92% after multiple tests, indicating that the parameters of the deep learning method set by the proposed method can meet the requirements of output accuracy. The convergence of the algorithm when the proposed method is randomly selected for single training is shown in Figure 7.

It can be seen from Figure 7 that when the data space samples are set, the proposed method has fast convergence speed, and the training accuracy can reach about 95% when the algorithm tends to be stable.

**Figure 8:**
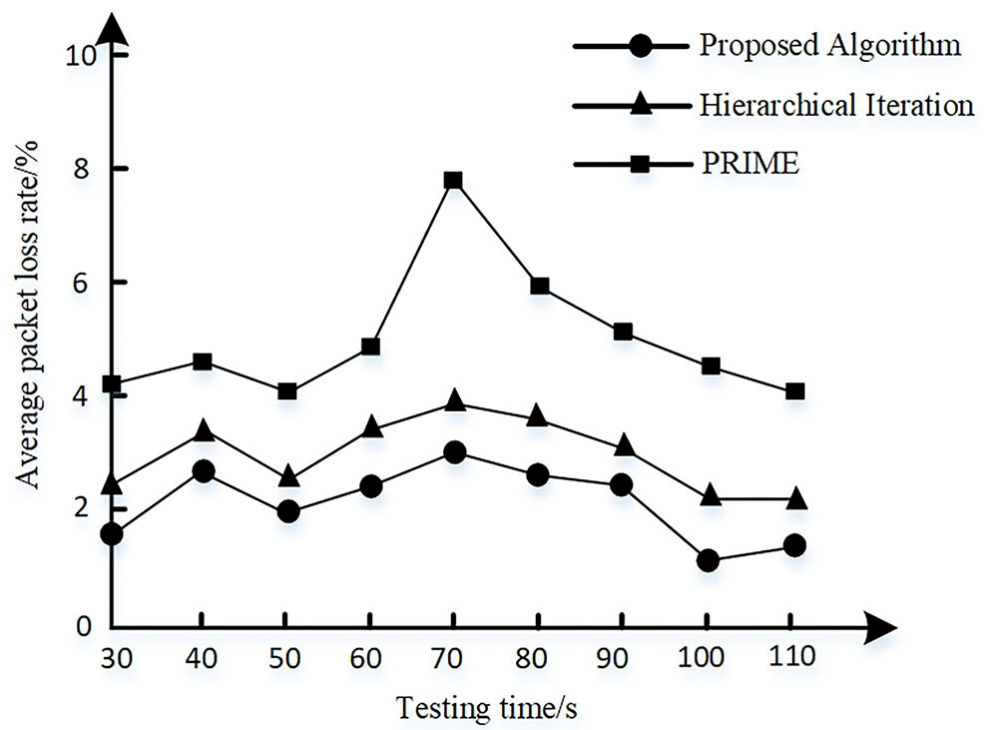
Average packet loss rate.

A 5G extranet host is set as the data sending node and the zero-trust platform is set as the data receiving node. The communication quality of each link is randomly set. The hierarchical iteration algorithm as provided in Hu et al. (2019) and powerline intelligent metering evolution (PRIME) algorithm as provided in Aruzuaga et al. (2010) are selected as the comparison method. The average packet loss rate of 5G power communication network is calculated where noise interference is randomly added to a link node at 60s. The results are shown in Figure 8.

It can be seen from Figure 8 that the average packet loss rate of the proposed algorithm is lower than the other two algorithms and is ~0.6% lower than the hierarchical iteration algorithm. When noise interference is added, the proposed and hierarchical iteration algorithm both can adjust adaptively and reduce the packet loss rate.

Second, through experiment 2, the performance of 5G power communication security system is tested and verified. The test results are shown in Table 1.

**Table 1:** Security performance test results of proposed system.

**Test items**	**Test results**
Latency from 5G extranet host to intranet host I	<100 ms
Latency from 5G extranet host to intranet host II	<90 ms
Authentication delay between 5G extranet host and zero-trust platform	<5 ms/time
Data encryption and decryption delay between 5G extranet host and zero-trust platform	<1 ms/time
Communication bandwidth between 5G extranet host and zero-trust platform (downlink)	>200 Mbps
Communication bandwidth between 5G extranet host and zero-trust platform (uplink)	>70 Mbps

As shown in Table 1, the authentication, encryption, and decryption delay between 5G extranet host and zero-trust platform is <6 ms each time, accounting for a small proportion in the overall communication delay. In the proposed system, the time delay mainly lies in the time delay of isolation facilities in the power system. Due to its data security filtering functions and technical architecture, the time delay of network security logical isolation device is greater than that of the forward and backward isolation devices. The overall bandwidth limitation in the proposed system mainly lies in the backward isolation device (Boyaci et al., 2022). The bandwidth between 5G extranet host and zero-trust platform can meet the large bandwidth requirements of video monitoring and other applications.

Finally, according to the security risks identified in 5G power communication scenario, the IXIA PerfectStorm ONE testbed is used to verify the security of the proposed system through experiment 3. The test results are shown in Table 2.

**Table 2:** Security test of the proposed system.

**Test items**	**Test results**
Malicious 5G terminal attempts to access	Access denied
Legitimate 5G terminal hijacked	Authority of terminal is degraded and the terminal is then disconnected
5G network data tampering	Failed
5G network data eavesdropping	Failed
Protocol fuzzy test	The system operates normally
DOS attack/200 Mbps	The system operates normally

According to Table 2, the proposed system can resist malicious terminal access, terminal hijacking, data tampering and eavesdropping, protocol fuzzy and DoS attacks, so as to reduce the security risk of 5G power communication.

In summary, experiment 1 verified the efficiency and performance of the intelligent link state algorithm, experiment 2 verified the secure communication performance of the proposed system, and experiment 3 verified the security of the proposed system. From these three experiments, it can be seen that the intelligent link state algorithm and improved multiple protection model proposed in this system demonstrated satisfied transmission efficiency and security performance, which may meet the demands of power 5G applications.

## Conclusions

In this study, a 5G power security system is proposed where an intelligent link state algorithm and an improved multiple protection model are designed. The intelligent link state algorithm is based on the deep learning method so as to suggest the optimal data transmission path between the 5G extranet host and the zero-trust security platform. The multiple protection model is improved *via* adopting the zero-trust architecture and CBR methodology. The details and operational process of the proposed system including link state algorithm and CBR algorithm are described. Three experiments are established to validate the efficiency and effectiveness of the proposed system. The future research directions may reside in the further improvement of the efficiency of the multiple protection model in the era of big data and IoT where millions of terminals will be connected.

## Data Availability Statement

The raw data supporting the conclusions of this article will be made available upon request to corresponding authors.

## Author Contributions

CJ and CH contributed to conception and design of the study. HX organized the database. QH performed the statistical analysis. CJ wrote the first draft of the manuscript. CJ, HX, CH, and QH wrote sections of the manuscript. All authors contributed to manuscript revision, read, and approved the submitted version.

## Funding

This research was supported by the Science and Technology Project of State Grid Jiangsu Electric Power Co., Ltd. (Grant No. J2021125), Jiangsu Provincial Social Science Foundation Youth Project (Grant No. 21TQC003), Major Project of Philosophy, and Social Science Research in Universities of Jiangsu Province Education Department (Grant No. 2020SJZDA069).

## Conflict of Interest

The authors declare that the research was conducted in the absence of any commercial or financial relationships that could be construed as a potential conflict of interest.

## Publisher's Note

All claims expressed in this article are solely those of the authors and do not necessarily represent those of their affiliated organizations, or those of the publisher, the editors and the reviewers. Any product that may be evaluated in this article, or claim that may be made by its manufacturer, is not guaranteed or endorsed by the publisher.
